# Hepatic progenitor cell activation is induced by the depletion of the gut microbiome in mice

**DOI:** 10.1002/mbo3.873

**Published:** 2019-05-16

**Authors:** Fei Wang, Nan‐nan Sun, Lan‐lan Li, Wan‐wan Zhu, Jianbo Xiu, Yan Shen, Qi Xu

**Affiliations:** ^1^ State Key Laboratory of Medical Molecular Biology, Institute of Basic Medical Sciences Chinese Academy of Medical Sciences, School of Basic Medicine Peking Union Medical College Beijing China; ^2^ Neuroscience center Chinese Academy of Medical Sciences Beijing China

**Keywords:** activation, deplete, gut microbiome, hepatic progenitor cell, panCK, Sox9

## Abstract

The homeostasis of the gut microbiome is crucial for human health and for liver function. However, it has not been established whether the gut microbiome influence hepatic progenitor cells (HPCs). HPCs are capable of self‐renewal and differentiate into hepatocytes and cholangiocytes; however, HPCs are normally quiescent and are rare in adults. After sustained liver damage, a ductular reaction occurs, and the number of HPCs is substantially increased. Here, we administered five broad‐spectrum antibiotics for 14 days to deplete the gut microbiomes of male C57BL/6 mice, and we measured the plasma aminotransferases and other biochemical indices. The expression levels of two HPC markers, SRY‐related high mobility group‐box gene 9 (Sox9) and cytokeratin (CK), were also measured. The plasma aminotransferase activities were not affected, but the triglyceride, lactate dehydrogenase, low‐density lipoprotein, and high‐density lipoprotein concentrations were significantly altered; this suggests that liver function is affected by the composition of the gut microbiome. The mRNA expression of Sox9 was significantly higher in the treated mice than it was in the control mice (*p* < 0.0001), and a substantial expression of Sox9 and CK was observed around the bile ducts. The mRNA expression levels of proinflammatory factors (interleukin [IL]‐1β, IL‐6, tumor necrosis factor [TNF]‐α, and TNF‐like weak inducer of apoptosis [Tweak]) were also significantly higher in the antibiotic‐treated mice than the levels in the control mice. These data imply that the depletion of the gut microbiome leads to liver damage, negatively impacts the hepatic metabolism and function, and activates HPCs. However, the underlying mechanisms remain to be determined.

## Graphical abstract

While many studies have investigated the liver pathology associated with gut microbiome dysbiosis. I have not come across a paper that deals with hepatic progenitor cells (HPC) activation and microbiome dysbiosis. We found depletion of gut microbiota could induce HPCs activation and the mechanism underlying this phenomenon may associate with inflammatory factors.

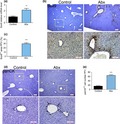

## 1 INTRODUCTION

The liver has numerous functions, including metabolism, bile secretion, glycogen storage, hematopoiesis, and immunity (Knell, 1980). The liver also detoxifies endogenous and exogenous substances, including medicines (Brockmoller & Roots, 1994), which may be harmful. However, liver pathology rarely results in clinical signs until the extent of the injury is severe; therefore, this organ should be carefully protected. The liver receives 70% of its blood supply from the gut *via* the portal vein (Krarup, Larsen, & Madsen, 1975), suggesting that this organ is the first to encounter absorbed nutrients, antigens, toxins, and microorganisms. The direct connection between the gut and the liver implies that the intestinal microbiome or its products will affect the liver.

The gut microbiome consists of more than 10^4^ species and ~10^14^ individual cells. The species and the number of cells of each species vary substantially in the intestine and according to host factors, including age, diet, and sex (Orrhage & Nord, 2000). The homeostasis of the gut microbiome plays an important role in physical health by affecting the functions of various visceral organs and may also affect mental health (Ivanov & Honda, 2012). Previous studies have revealed that a wide variety of hepatic diseases are associated with disturbances in the gut microbiome, such as nonalcoholic steatohepatitis, cirrhosis, alcoholic liver cirrhosis, and hepatic carcinoma (Bajaj et al., 2012; Chen et al., 2011; Fukui, Brauner, Bode, & Bode, 1991; Imajo et al., 2012; Lu et al., 2011; Qin et al., 2014; Rivera, Bradford, Seabra, & Thurman, 1998; Tuomisto et al., 2014). For instance, alcoholic liver disease is associated with the perturbation of the gut‐liver axis because of large quantities of lipopolysaccharide (LPS), peptidoglycan, bacterial 16S DNA, and other deleterious bacterially derived molecules entering the liver. This perturbation results in macrophage (Kupffer cell) activation and the secretion of inflammatory cytokines, including interleukin (IL)‐6 and IL‐1β, and reactive oxygen species; this, in turn, increases the permeability and inflammation of the gut, creating a vicious circle. In addition, the activation of stellate cells results in the increased expression of α‐smooth muscle actin and collagen, which contribute to liver fibrosis and cirrhosis (Szabo, 2015).

Studies of hepatic progenitor cells (HPCs) have significantly progressed in recent years. As early as 1958, Wilson and Leduc identified bipotential liver progenitor cells that could differentiate into hepatic parenchymal cells or into biliary epithelial cells (Wilson & Leduc, 1958). By altering the culture conditions of these cells, several laboratories have been able to replicate this bidirectional differentiation in vitro (Furuyama et al., 2011; Hao et al., 2013; Huch et al., 2013; Li et al., 2006; Suzuki et al., 2008). HPCs account for <1% of the total number of liver cells, with almost all of these cells being dormant and located around the Hering ducts. However, when the liver is subjected to sustained damage, such as that resulting from the intraperitoneal injection of carbon tetrachloride (Petersen, Zajac, & Michalopoulos, 1998), bile duct ligation (Irie et al., 2007; Mu et al., 2017; Wu, Ma, Gibson, Hirai, & Tsukada, 1981), choline‐deficient and ethionine‐supplemented diet (Akhurst et al., 2001; Passman et al., 2015), methionine‐choline‐deficient diet (Morell et al., 2017), thioacetamide diet or partial hepatectomy (Chien et al., 2018; Grzelak et al., 2014; Kohn‐Gaone et al., 2016; Lu et al., 2016), HPCs are stimulated to proliferate and to protect and regenerate the injured liver. The source of HPCs remains controversial. Some researchers contend that these cells exist in a quiescent state around bile ducts until activated (Tee, Kirilak, Huang, Morgan, & Yeoh, 1994; Tee, Smith, & Yeoh, 1992), while other researchers contend that some HPCs originate from hepatocytes (Wu & Lee, 2017) or from an extrahepatic source, especially bone marrow (Li et al., 2011; Zhai et al., 2018). In addition, recent studies have indicated that HPCs originate from the transdifferentiation of biliary epithelial cells (Raven et al., 2018). However, it has not been established whether the disturbance of the gut microbiome influences HPC activation.

In this study, we treated C57BL/6 mice with five broad‐spectrum antibiotics for 14 days at the maximum dose to eliminate their gut microbes. Subsequently, we assessed the serum biochemical indices and quantified the expression of the progenitor cell marker SRY‐related high mobility group‐box gene 9 (SOX9) (Lo, Chan, Leung, & Ng, 2018; Pozniak et al., 2017; Tarlow, Finegold, & Grompe, 2014). The plasma aspartate aminotransferase (AST) and alanine aminotransferase (ALT) activities were not significantly higher in the treated mice than they were in the control mice, but the triglyceride (TG), lactate dehydrogenase (LDH), low‐density lipoprotein (LDL), and high‐density lipoprotein (HDL) concentrations were all affected. In addition, the expression levels of SOX9 (*p* < 0.0001) and the progenitor cell marker cytokeratin (CK) (Russell et al., 2019) were significantly higher in the livers of the antibiotic‐treated mice than their expression levels in the control mice. The mechanism of these effects may involve the induction of inflammation because the expression levels of proinflammatory factors (IL‐1β, IL‐6, tumor necrosis factor (TNF)‐α, and TWEAK) were also higher. These results indicate that the elimination of the intestinal flora impairs liver function and activates HPCs. These findings add to our understanding of gut‐liver and microbiome‐liver interactions and of the regulation of HPC activation.

## 2 RESULTS

### 2.1 Depletion of the gut microbiome results in cecal enlargement and increased colonic permeability

**Figure 1:**
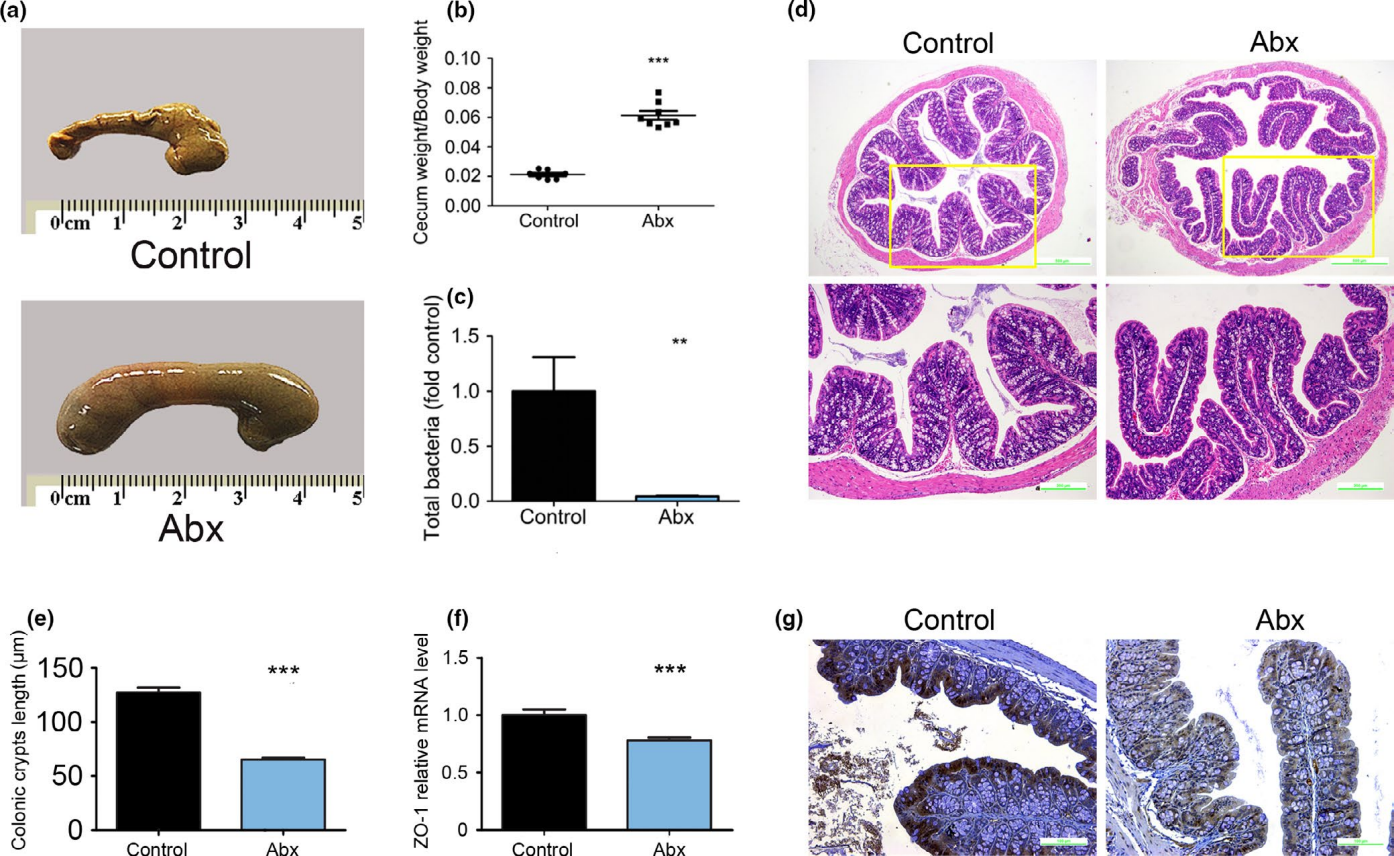
Cecal enlargement is induced by the maximum depletion of the gut microbiome. (a) Representative images of the cecum in control and Abx mice. (b) Cecal mass/body mass in control and Abx mice. (c) 16S rRNA analysis of Abx and control mice. (d) Representative colonic H&E‐stained images. (e) Colonic crypt length comparison. (f) Tight junction protein ZO‐1 relative mRNA level. (g) Representative immunochemistry images of ZO‐1 expression. In all panels, the data are expressed as the mean ± SEM; control (*n* = 8), Abx (*n* = 8); **p < 0.01 versus control, ***p < 0.0001 versus control. H&E, hematoxylin and eosin

To deplete the intestinal microbiome, male C57BL/6 mice were treated with an antibiotic cocktail containing meropenem, neomycin sulfate, natamycin, bacitracin, and vancomycin in their drinking water for 14 days (Abx mice). The selected antibiotics do not reach the liver *via* enterohepatic circulation, which excludes the possibility of direct effects on the liver (Frohlich et al., 2016; Guida et al., 2018; Heimesaat et al., 2006; Mohle et al., 2016). The ceca of the Abx mice were obviously larger than those of the control mice (Figure 1a), such that the ratio of cecal mass to body mass of the Abx mice was substantially higher than that of the control mice (Figure 1b). This cecal enlargement suggests that the gut microbiome plays an important role in the maintenance of intestinal morphology and health. Next, 16S rDNA quantitative polymerase chain reaction (qPCR) was also performed (Figure 1c), which demonstrated that the vast majority of gut microbes had been eliminated. Colonic hematoxylin and eosin (H&E) staining showed that the crypt length was obviously decreased and that the number of goblet cells was largely reduced (Figure 1d,e). Goblet cells secrete the mucus layer, which protects the colonic epithelium from the contents of the lumen. The poor changes in crypt length and goblet cell number indicated that the colonic epithelium was damaged. To further confirm this finding, the tight junction protein ZO‐1 was analyzed by real‐time PCR and immunochemistry (Figure 1f,g). Both the mRNA and protein levels were significantly reduced in the Abx mice compared to those in the control mice.

### 2.2 Depletion of the gut microbiome causes liver dysfunction

**Figure 2:**
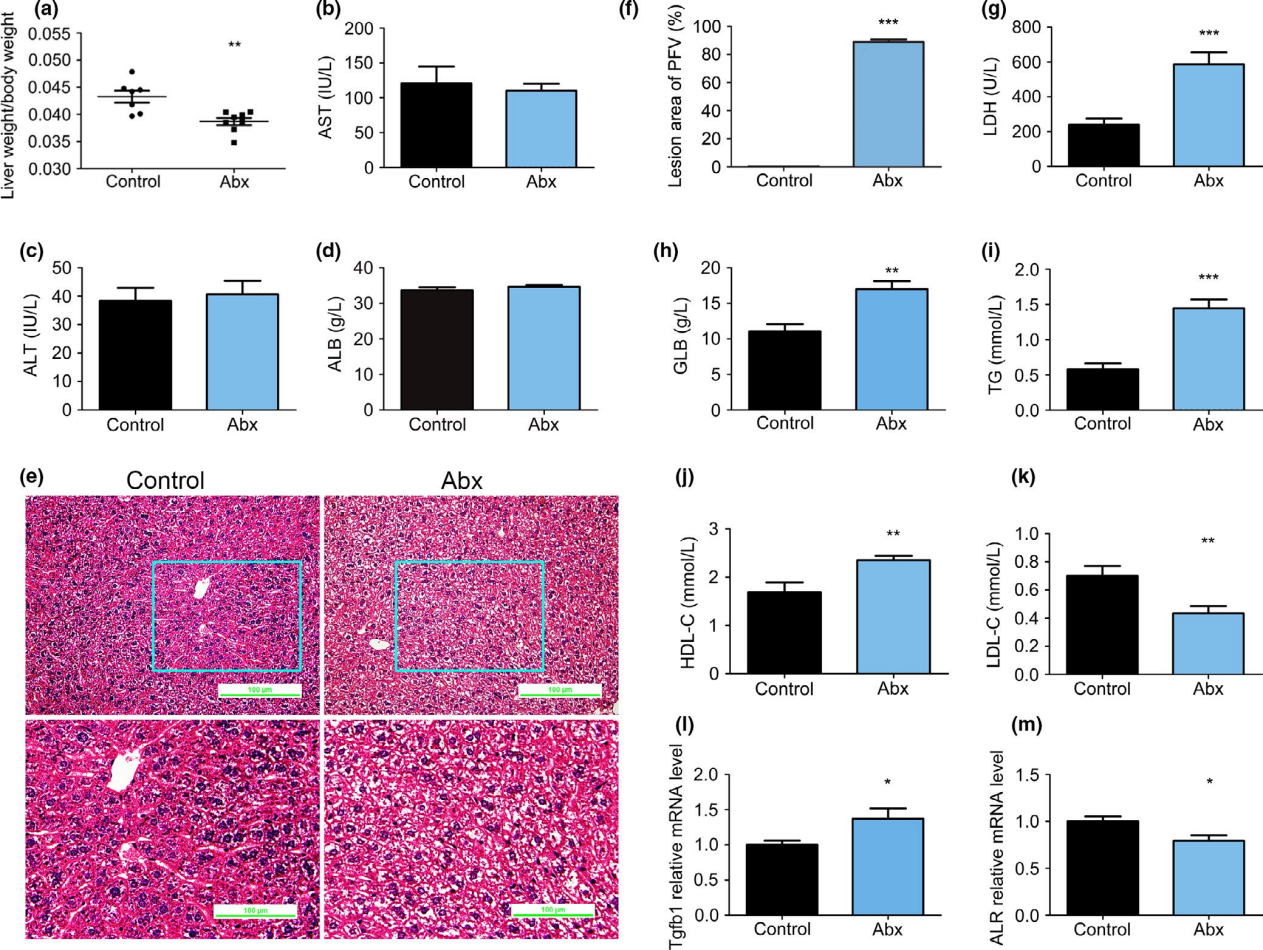
Serum biochemical indices and hematoxylin‐eosin (H&E) staining in control and Abx mice. (a) Liver mass/body mass in control and Abx mice. Serum AST (b), ALT (c), and ALB (d) in control and Abx mice. (e) Representative images of H&E staining in control and Abx mice. (f) The lesion area was estimated using PFV analysis of the H&E‐stained images. Serum LDH (g), GLB (h), TG (i), HDL‐C (j), and LDL‐C (k) concentrations in control and Abx mice. (l) Tgfb1 mRNA expression in control and Abx mice. (m) ALR mRNA expression in control and Abx mice. In all panels, the data are expressed as the mean ± SEM; control (*n* = 8), Abx (*n* = 8); *p < 0.05 versus control, **p < 0.01 versus control, ***p < 0.0001 versus control. ALB, albumin; ALT, alanine aminotransferase; AST, aspartate aminotransferase; GLB, globulin; HDL, high‐density lipoprotein; LDH, low‐density lipoprotein; LDL, low‐density lipoprotein; TG, triglyceride

The maximum depletion of the gut microbiome caused a significant reduction in the ratio of liver mass to body mass (Figure 2a). Serum AST and ALT did not differ between the Abx and control mice (Figure 2b,c), implying that hepatic parenchymal cells were not lysed as a result of antibiotic treatment. Plasma albumin (ALB) was also unaffected (Figure 2d). However, H&E staining of liver sections showed hepatocytomegaly, cytoplasmic rarefaction, and a loss of visible hepatic cord structure (Figure 2e,f). In addition, serum LDH, globulin (GLB), TG, HDL‐C, and LDL‐C were all significantly affected by antibiotic treatment (Figure 2g–k), implying the presence of hepatic pathology, inflammation, and disordered lipid and cholesterol metabolism.

Transforming growth factor‐beta 1 (TGFB1) is one of the three subtypes of TGF‐β, a multifunctional protein that regulates cell growth, differentiation, apoptosis, the immune response and cellular homeostasis (Heldin, Miyazono, & Ten, 1997; Seoane & Gomis, 2017; Shi & Massague, 2003; Wu & Hill, 2009), and hence, can be regarded as a marker of hepatocyte dysfunction (Raven et al., 2018). Higher *Tgfb1* expression was found in the livers of Abx mice (Figure 2l). Augmenter of liver regeneration (ALR) stimulates hepatocyte proliferation, protects the liver from injury, and participates in organ formation and development (Gandhi, 2012; Hongbo et al., 2012; Mu et al., 2016; Vodovotz et al., 2013), and the ALR mRNA expression level was lower in the Abx mice than its expression in the control mice (Figure 2m). These results imply that liver dysfunction and morphological changes result from gut microbial ablation.

### 2.3 Depletion of the gut microbiome in adult mice activates HPCs

**Figure 3:**
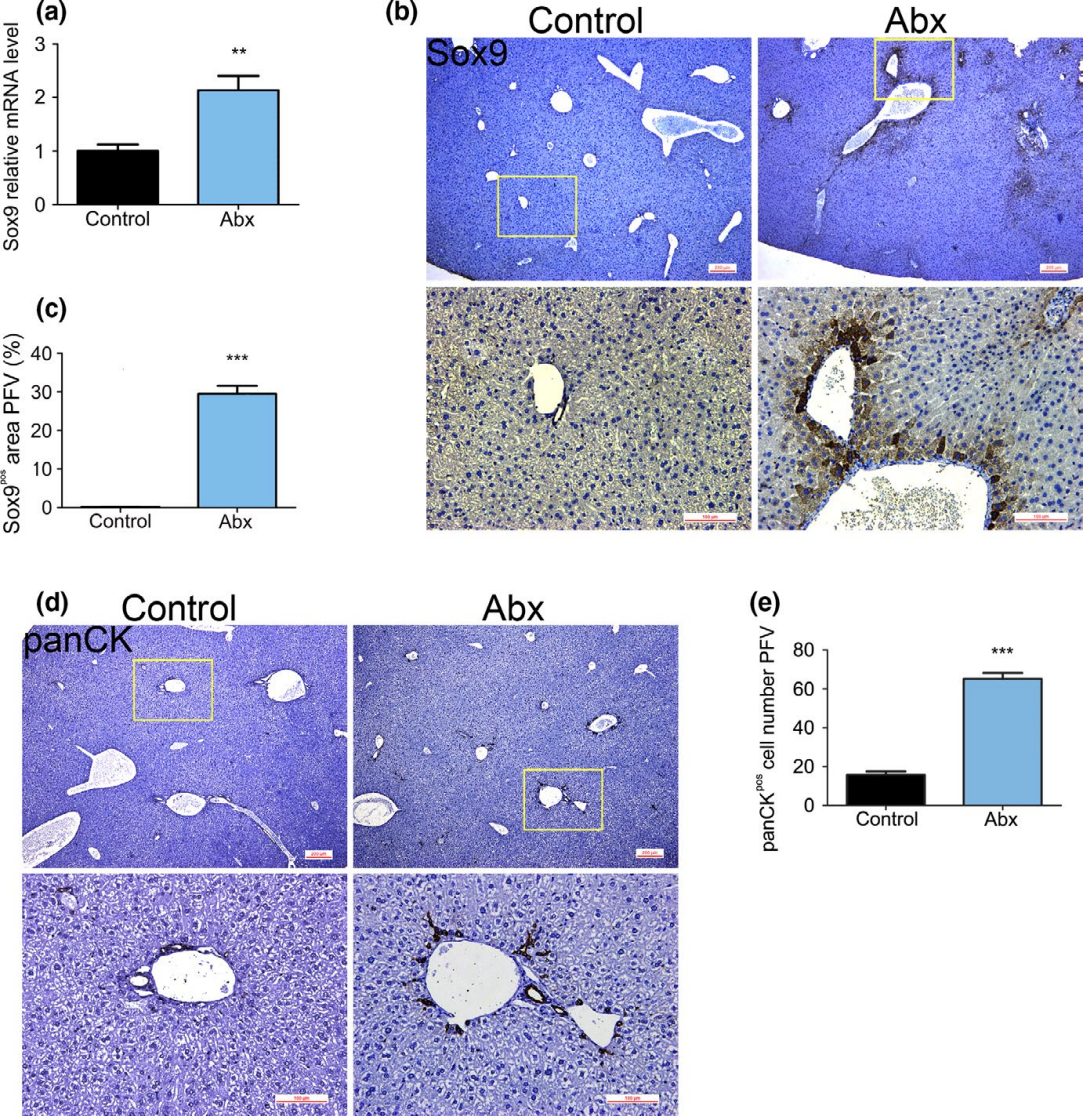
The expression levels of the hepatic progenitor cell markers SOX9 and CK were significantly increased by microbiome ablation. (a) *Sox9* mRNA expression was determined by qPCR to be significantly higher in Abx mice than in control mice. (b) Representative immunohistochemical staining for SOX9 in control and Abx mice. (c) The SOX9‐positive area in the control and Abx mice was estimated using PFV analysis. (d) Representative immunohistochemical staining for pan‐CK in control and Abx mice. (e) The pan‐CK‐positive cell number in the control and Abx mice was estimated using PFV analysis. Control (*n* = 8), Abx (*n* = 8); **p < 0.01 versus control, ***p < 0.0001 versus control. CK, Cytokeratin; PFV, per field of view; qPCR, quantitative polymerase chain reaction

SOX9 is important in early embryonic development and is a member of a family of genes homologous to the sex‐determining region of the Y chromosome (*Sry*). The *Sry* gene induces the development of mammalian testes and participates in gender determination. In previous studies, SOX9 has been used as a progenitor or stem cell marker (Hongbo et al., 2012; Tanimizu, Nishikawa, Ichinohe, Akiyama, & Mitaka, 2014). The mRNA expression of *Sox9* was significantly higher in the livers of Abx mice (Figure 3a) than it was in the livers of the control mice, suggesting that HPCs were activated. SOX9 immunohistochemistry (Figure 3b) showed that this protein was expressed around the bile ducts in the Abx mice, whereas in the control mice, there were very few SOX9‐expressing cells. The quantification of the SOX9‐positive area per field of view (PFV) is shown in Figure 3c.

Cytokeratins are a group of intermediate filament proteins that are expressed in both keratinized and nonkeratinized epithelial tissues. These proteins maintain the overall structural integrity of epithelial cells and play a crucial role in tissue differentiation and specialization. CK expression has therefore been used as a marker of tissue differentiation, for example, to assess tumor malignancy (Barak, Goike, Panaretakis, & Einarsson, 2004; Moll, 1998; Nicolini, Ferrari, & Rossi, 2015) and progenitor cell activation. Immunostaining using a pan‐CK antibody showed that CKs were prominently expressed in the Abx mouse liver but were not expressed in the control mouse liver (Figure 3d). The estimated number of pan‐CK‐positive cells PFV is shown in Figure 3e. In contrast to the situation of SOX9, a percentage of the stained area was not used to quantify CK expression because in the pan‐CK‐stained sections, positive cells were present in well‐defined borders. These data demonstrate that HPCs are activated following gut microbial depletion.

### 2.4 Depletion of the gut microbiome is associated with macrophage activation and the higher expression of genes encoding proinflammatory cytokines in the liver

**Figure 4:**
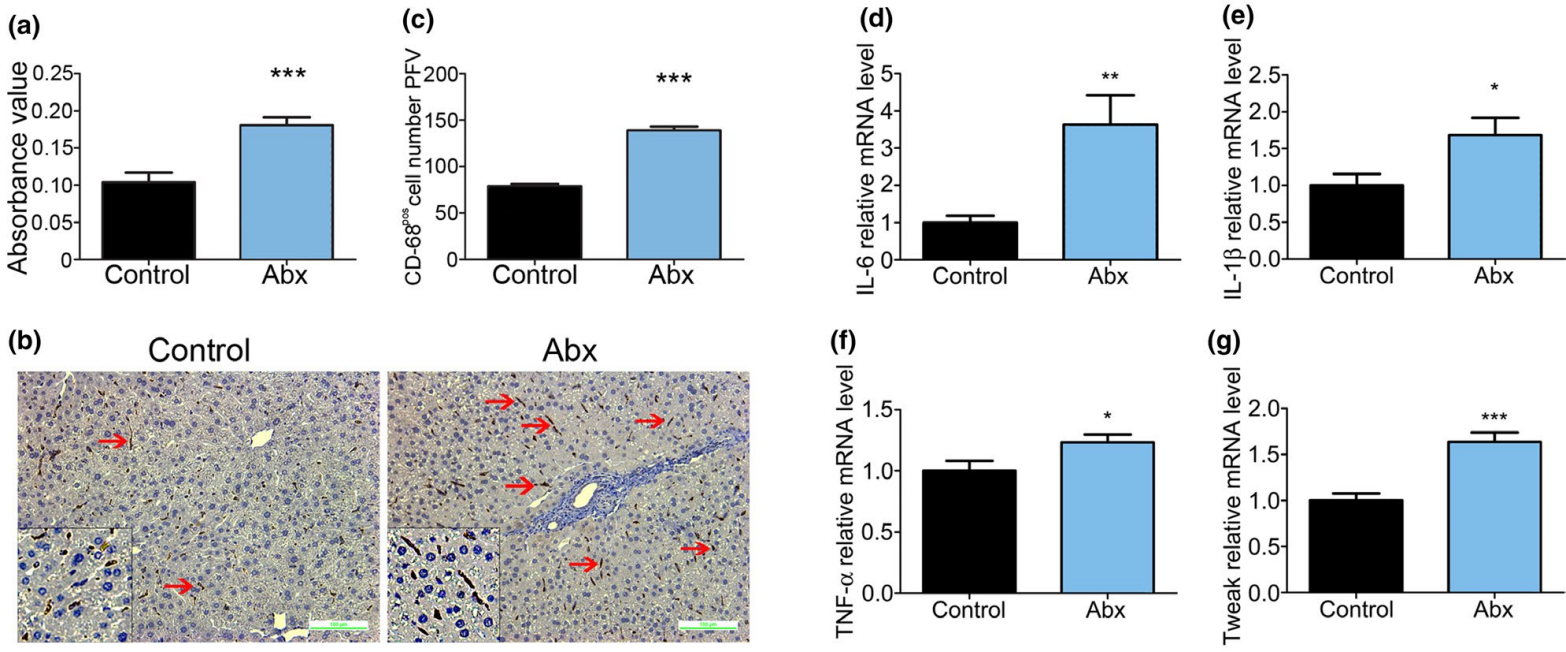
Inflammatory cytokine expression is much higher in the Abx mice than it is in the control mice. (a) Serum LPS ELISA absorbance value comparison. (b) Representative images of CD68 immunochemistry. Each red arrow indicates a CD68‐positive macrophage. The bottom left corner is the enlarged image, and the brown corners are macrophages. (c) CD68‐positive cell number PFV in control and Abx mice. (d) *IL‐6* mRNA expression was determined by real‐time PCR to be significantly higher in the Abx mice than it was in the control mice. (e) *IL‐1β* mRNA expression was much higher in the Abx mice than that in the control mice. (f) *TNF‐α* mRNA expression was much higher in the Abx mice than that in the control mice. (g) *Tweak* mRNA expression was determined using qPCR to be significantly higher in the Abx mice than that in the control mice. Control (*n* = 8), Abx (*n* = 8); *p < 0.05 versus control, **p < 0.01 versus control, ***p < 0.0001 versus control. PFV, per field of view; qPCR, quantitative polymerase chain reaction; LPS, lipopolysaccharide

We next explored the mechanism underlying HPC activation following antibiotic treatment. Following antibiotic treatment, the serum LPS level was significantly increased in the Abx mice compared to that in the control mice (Figure 4a). According to the literature (Bawa & Saraswat, 2013; Malaguarnera, Giordano, Nunnari, Bertino, & Malaguarnera, 2014; Szabo, 2015; Yang et al., 2019), in the gut‐liver axis, LPS is a key component that binds to the toll‐like receptor (TLR) family members to activate macrophages, which are associated with several liver diseases. Because CD68 is a marker of macrophages, we analyzed CD68 protein expression by immunochemistry (Figure 4c) and counted the number of marked macrophages (Figure 4b). The number of macrophages in the Abx mice was obviously increased compared with that of the control mice. We hypothesized that proinflammatory cytokines might contribute to HPC activation. IL‐6 is a pluripotent cytokine that is principally secreted by macrophages during the acute phase response, inflammation, bone catabolism, hematopoiesis, and cancer progression. IL‐6 could therefore represent a biomarker of disease severity and a prognostic indicator (Ji et al., 2016; Rincon, 2012). The expression of *IL‐6* was higher than that of *TNF‐α* and *IL‐1β* in the mice and was significantly higher in the Abx mice than it was in the control mice (Figure 4d, *p* < 0.01). IL‐1β, which is secreted by macrophages, is an important mediator of inflammation and has other functions, including cell proliferation, differentiation, and apoptosis (Chung et al., 2015; Sato et al., 2014; Shrivastava, Mukherjee, Ray, & Ray, 2013). IL‐1β expression was also higher in the Abx mice than it was in the control mice (Figure 4b). TNF‐α, a cytokine that is produced mainly by activated macrophages, is involved in the systemic inflammatory response and contributes to the acute phase response (Ji et al., 2016; Sato et al., 2014). The mRNA expression of *TNF‐α* in the Abx mice was higher than that in the control mice (Figure 4c). TWEAK is a cytokine produced by macrophages, belongs to the TNF superfamily and may regulate inflammation, cell apoptosis, and proliferation. TWEAK has been shown to be associated with HPC activation in previous studies (Akahori et al., 2015; Hamill, Michaelson, Hahm, Burkly, & Kessler, 2007; Sheng et al., 2018). The mRNA expression of *Tweak* was significantly higher in the Abx mice than it was in the control mice (Figure 4d, *p* < 0.0001). These data may imply that macrophages play a critical role in HPC activation by releasing inflammatory cytokines, especially IL‐6 and TWEAK. Thus, these inflammatory cytokines may be regarded as key targets for more extensive research.

## 3 DISCUSSION

The depletion of the gut microbiome led to a reduction in liver mass, suggesting that liver function might be impaired. Although the serum ALT and AST activities and the ALB concentration were not affected, H&E staining demonstrated that hepatocytomegaly, cytoplasmic rarefaction, and a loss of hepatic cord structure had occurred, implying liver cell damage. Lipid metabolism (as shown by TG), cholesterol metabolism (as shown by LDL and HDL), glycolysis (as shown by LDH), and inflammatory status (as shown by GLB), were all substantially affected by the depletion of the gut microbiome, confirming hepatocyte dysfunction. Most importantly, we found evidence that HPCs were activated because the progenitor cell markers SOX9 and CK were both expressed at higher levels. Inflammatory cytokines, especially IL‐6 and TWEAK, may mediate this effect.

The gut microbiota and its human host have a symbiotic relationship once the microbiome has been gradually generated and stabilized during the first 1–3 years of life (Milani et al., 2017; Tanaka & Nakayama, 2017). Various hepatic diseases have been shown to be linked to gut microbial dysbiosis, including nonalcoholic fatty liver disease, alcoholic liver disease, hepatic fibrosis, sclerosis, and hepatic carcinoma. The liver receives two blood supplies: one‐quarter comes from the systemic circulation *via* the hepatic artery, and the other three‐quarters arrive *via* the portal vein from the gut. These two blood supplies suggest that the liver is the first organ to encounter nutrients, antigens, toxins, and other substances absorbed from the gut. This connection has been termed the “gut‐liver axis,” and its importance in the pathogenesis of liver diseases has been repeatedly shown since it was first described in 1998 (Seabra, Stachlewitz, & Thurman, 1998).

Most of the deleterious effects of gut microbes on the liver are mediated through inflammation (Bawa & Saraswat, 2013; Yamada et al., 2017), which is triggered by interactions between the microbes, the liver, and the immune system. Key to this process is the interaction of hepatic macrophages (Kupffer cells) with pathogen‐associated molecular patterns (PAMPs). PAMPs, especially endotoxin or LPS, bind to TLRs (Miura & Ohnishi, 2014; Miyake & Yamamoto, 2013), particularly to TLR4 (Carotti, Guarino, Vespasiani‐Gentilucci, & Morini, 2015), and activate Kupffer cells, which attempt to remove pathogens from the liver. As our results show, the depletion of the gut microbiota resulted in massive reductions in goblet cells and significantly shortened the crypt lengths. The expression of the tight junction protein ZO‐1 was decreased in the Abx mice compared to that in the control mice. These results indicate that gut permeability was increased, allowing LPS to penetrate the gut wall and be absorbed into the bloodstream. In line with the above results, the concentration of LPS in the serum was significantly higher in the Abx mice compared to that in the control mice. Moreover, macrophages in the livers of Abx mice were substantially activated. These observations were consistent with previous literature reports that have indicated that LPS activates macrophages (Ghosh, Bie, Wang, & Ghosh, 2014). To achieve the removal of pathogens, a series of inflammatory pathways are activated, and a number of cytokines are released. In addition, hepatic stellate cells express TLR4 and are therefore sensitive to LPS. Once these cells are activated by LPS, they produce larger amounts of collagen, which promotes fibrosis.

The greatest current challenge in research on the gut microbiome is to determine whether it plays a causative role in physiological and pathological processes. The current experimental strategies make use of pathogen‐free rodents, antibiotic‐induced microbial modifications, infection with enteric pathogenic bacteria, and transplantation with probiotics or fecal flora. In pathogen‐free mice, fecal bacterial transplantation can be used to study the role of the microbiome in a disease process or to assess the effects of the gut microbial composition during development (Lundberg, Toft, August, Hansen, & Hansen, 2016). Given that it is impossible to use this approach in humans, the standard approach remains a combination treatment with multiple antibiotics to ablate the gut microbiome. Antibiotic treatment can be used to control the composition of the intestinal flora in a controlled, clinically feasible way to study the effects of particular types of intestinal microorganisms on the host.

Under normal circumstances, very few HPCs are evident in the liver. This finding contrasts with the situation in many other organs and tissues, such as the intestine and hair follicles, in which progenitor cells continuously divide and supply new daughter cells. Under normal circumstances, adult liver parenchymal cells remain in the G0 phase. However, when the liver suffers a major insult, such as hepatectomy or toxic damage, the main source of daughter cells tends to be the hepatic parenchymal cells. Only if the division of hepatic parenchymal cells is inhibited, such as during chronic liver failure, are HPCs activated, and these cells proliferate and differentiate to generate new hepatic parenchymal cells and cholangiocytes. In this study, the depletion of the gut microbiome not only negatively affected liver health but also induced a ductular reaction; and the progenitor markers SOX9 and CK were expressed in larger quantities around the lumen.

Interleukin‐6 is a cytokine that has multiple effects and redundant activities. IL‐6 is mainly produced by macrophages in response to PAMPs and damage‐associated molecular patterns (Jinkawa et al., 2019); IL‐6 plays a role in the removal of infectious agents during the acute phase and during immune responses to protect and repair the damaged tissue (Gonzalez et al., 2018; Mendlovic et al., 2017). IL‐6 can be used as a biomarker of disease severity and as a prognostic indicator (Jang et al., 2012); in addition, its expression is always higher than that of TNF‐α and IL‐1β. IL‐6 has traditionally been considered an obvious proinflammatory cytokine. IL‐6 is involved in the development of tumors associated with inflammation and the regulation of innate and/or adaptive immune responses (Chen et al., 2018; Giardino et al., 2017). In addition to its deleterious effects on inflammation and cancer, IL‐6 has been shown to have a regenerative and antiinflammatory potential (Naseem, Hussain, & Manzoor, 2018).

Transmembrane IL‐6 receptor expression was limited to the surface of liver cells and to some leukocytes. Another molecule, called soluble IL‐6 receptor (sIL‐6R), is present in the tissue fluid and serum and makes it possible to combine IL‐6 with many other cell types (Tanaka & Kishimoto, 2014). This allows IL‐6 to be delivered to the cells that do not express the IL‐6 receptor. The IL‐6/IL‐6R complex binds to the signal transducer gp130 to activate the Janus Kinase (JAK) and the signal transducer and activator of transcription 3 (STAT3) pathways (Kubo, Hanada, & Yoshimura, 2003). The activation of the mitogen‐activated protein kinase (MAPK) signaling pathway in rapidly proliferating cells is usually observed after IL‐6 stimulation (2003, Jones, Horiuchi, Topley, Yamamoto, & Fuller, 2001), and IL‐6 signaling directly activates the prosurvival phosphatidylinositol 3‐kinase/Akt pathway (Levy & Lee, 2002).

Although IL‐6 is associated with many liver diseases and cancers, it is also crucial for liver regeneration (Naseem et al., 2018). IL‐6 mediates various pathways in the liver, including immediate early gene transcription, DNA repair, glycogen storage, antioxidant activity, angiogenesis, cell proliferation, and liver protection. IL‐6R and gp130 are widely expressed in all liver cells, including Kupffer cells, hepatic stellate cells, hepatocytes, and sinus endothelial cells. IL‐6 has been shown to regulate HPCs (Lu et al., 2015). By IL‐6/STAT3 signal transduction, HPCs proliferated and supported liver regeneration, while the absence of IL‐6 would limit the proliferation of HPCs and reduce liver regeneration (Yeoh et al., 2007). In addition, the number of HPCs in IL‐6‐deficient mice decreased, and in the absence of IL‐6 signaling, more liver failure with cell necrosis and impaired regeneration occurred (Cressman et al., 1996).

TWEAK, a member of the TNF superfamily, is expressed in multiple tissues. The many biological functions of TWEAK include the regulation of cell apoptosis and necrosis, proliferation, migration, and differentiation; in addition, this protein can trigger angiogenesis and induce the expression of inflammatory cytokines (Xu, Zhao, & Liu, 2016). TWEAK has only one receptor, fibroblast growth factor‐inducible 14 (Fn14), which is a type I transmembrane protein, and TWEAK is the only ligand of Fn14. TWEAK is widely expressed in dendritic cells, monocytes, macrophages, and natural killer cells (Liu, Xiao, & Xia, 2017). Among inflammatory cells, macrophages and monocytes are the main sources of TWEAK, while in normal tissues, the expression levels of TWEAK and Fn14 are very low. Higher levels of TWEAK and Fn14 expression usually appear in response to stress, tissue damage, or remodeling; this expression can also occur in connection with many other disorders, including autoimmune diseases and cancers (Gu et al., 2013; Wang et al., 2017), triggering the activation of multiple downstream signaling pathways that can modify these tissues.

Jakubowski et al. (2005) suggested that TWEAK is a direct mitogen of HPCs. Fn14 has no intrinsic protein kinase activity and is associated with tumor necrosis factor receptor–associated factor receptor molecules to trigger activated nuclear factor kappa light chain enhancer of activated B cells (NFκB) (Tirnitz‐Parker et al., 2010); extracellular signaling regulates the kinase and MAPK pathway of c‐Jun N‐terminal kinase. Feng et al. (2000) reported a rapid induction of Fn14 expression in early liver regeneration after partial hepatectomy. The authors also studied hepatocellular carcinoma cell lines, where the overexpression of Fn14 occurred in poorly differentiated cell lines. This suggests that it may be linked to progenitor cells. Jakubowski et al. confirmed this hypothesized relationship between HPCs and Fn14 signaling pathways with experiments. Their study showed that TWEAK overexpression by the transgenic or adenovirus methods can induce the HPC response. In the mouse model of oval cell (another name for HPCs in rodents) proliferation, TWEAK/Fn14 knockout inhibited the appearance of HPCs. In addition, TWEAK can directly induce the proliferation of bile duct epithelial cell lines with progenitor characteristics (Liu et al., 2015).

Because all of these inflammatory cytokines can be secreted by macrophages, these cells are likely to play a central role in the activation of HPCs in response to gut microbial depletion. We may speculate that with the removal of intestinal bacteria, intestinal homeostasis is disrupted and permeability is increased, causing LPS to leak into the blood. Excessive LPS activates the macrophages in the liver. Activated macrophages secrete inflammatory cytokines, especially IL‐6 and TWEAK. One pathway is that IL‐6 binds to IL‐6R and then binds to gp130, thereby activating the JAK/STAT 3 signaling pathway and activating the HPC response; the other pathway is that TWEAK binds to Fn14 to activate NFκB, thereby activating the HPC response. However, the specific mechanisms need to be defined, and there may be other mechanisms involved.

To our knowledge, our study is the first to demonstrate a link between the gut microbiome and HPC activation. We have demonstrated that the elimination of the vast majority of intestinal microbes can stimulate HPC activation, suggesting that HPC activity may be triggered by microbial dysbiosis. The mechanism underlying this phenomenon may involve the release of proinflammatory cytokines, especially IL‐6 and TWEAK, with macrophages playing a major role during this process. Further work is necessary to illuminate the precise mechanisms.

## 4 MATERIALS AND METHODS

### 4.1 Mice

Male C57BL/6 mice were purchased from Beijing Vital River Laboratory (China). According to the national standard (GB 14925‐2001), we housed the specific‐pathogen‐free grade mice in a barrier system under strict microorganism control and a controlled 12‐hr light‐dark cycle. Eight‐week‐old mice were used in the experiments.

The cocktail of antibiotics used consisted of neomycin (2 mg/ml, neomycin sulfate, HY‐B0470, MedChem Express [MCE]), meropenem (1 mg/ml, meropenem Trihydrate, M2279, J&K Scientific, China), bacitracin (5 mg/ml, 184862, J&K Scientific), vancomycin (1 mg/ml, vancomycin hydrochloride extracted from *Streptomyces orientalis*, 122263, J&K Scientific), and natamycin (1.2 μg/ml, HY‐B0133, MCE), which were all dissolved in autoclaved water (Frohlich et al., 2016; Guida et al., 2018; Kiraly et al., 2016) and were included in the drinking water of the mice for 14 days to ablate the gut microbiome. At the end of the experiment, the mice were anesthetized with 5% chloral hydrate, blood was collected from the retro‐orbital sinus, and then the mice were sacrificed by cervical dislocation. The ceca and livers were collected. A portion of each liver was snap‐frozen in liquid nitrogen for subsequent qPCR, and another portion was immersed in 4% paraformaldehyde for subsequent paraffin embedding. The cecal samples were snap‐frozen and then placed in a −80°C freezer. The blood samples were centrifuged for 10 min at 1,000×*g*, and then the serum samples was collected and delivered at 4°C to the Capital Medical University Clinical Laboratory Center for the assays.

### 4.2 LPS ELISA analysis

The ELISA Kit for LPS was purchased from Cloud‐Clone Corp. According to the instruction manual, 100 μl each of the standard dilution, blank and serum samples was added to the appropriate wells. The plate was covered with a sealer and was incubated at 37°C for 1 hr. Then, the liquid was removed, and 100 μl of Detection Reagent A was added to each well; the plate was covered and incubated at 37°C for 1 hr. Subsequently, the liquid was discarded, and 350 μl of 1x Wash Solution was added to each well followed by a 1–2 min incubation. The wash step was repeated three times. The plate was turned upside down and tapped onto absorbent paper to remove the excess liquid. Then, 100 μl of Detection Reagent B was added to each well, and the plate was covered and incubated for 30 min at 37°C. The abovementioned washing process was repeated five times. Subsequently, 90 μl of Substrate Solution was added to each well, and the plate was covered and incubated in the dark at 37°C for 10–20 min until the standard liquid turned blue. Next, 50 μl of Stop Solution was added to each well to terminate the reaction, and the liquid turned yellow. The bubbles on the surface of the liquid were avoided, and the absorbance was immediately measured at 450 nm.

### 4.3 Mouse cecal DNA isolation and 16S rRNA sequencing analysis

Metagenomic DNA was extracted from the cecal contents using the Powersoil DNA Isolation Kit (MoBio) according to the manufacturer's instructions. Then, 0.25 g of the cecal contents was added to the PowerBead tubes and was gently vortexed. Next, 60 μl of Solution C1 was added to the tubes and was centrifuged at 10,000×*g* for 30 s at room temperature (RT). A new 2 ml collection tube was utilized to collect the supernatant. Subsequently, 250 μl of Solution C2 was added, followed by vortexing and incubation at 4°C for 5 min. After centrifugation at 10,000×*g* for 1 min at RT, 500 μl of the supernatant was transferred to a new 2 ml collection tube. Then, 200 μl of Solution C3 was added, followed by vortexing, incubation at 4°C for 5 min and centrifugation at 10,000×*g* for 1 min at RT. Subsequently, 700 μl of supernatant was transferred to a new 2 ml collection tube, and 1,200 μl of Solution C4, which was shaken before use, was added to the supernatant and vortexed slightly. Each sample was processed by loading three times (675 μl each) onto a spin filter and centrifuging at 10,000×*g* for 1 min at RT; the flow through was discarded every time. Next, 500 μl of Solution C5 was added, followed by centrifuging at 10,000×*g* for 30 s at RT, discarding the flow through, and centrifuging again at 10,000×*g* for 1 min at RT. The spin filter was carefully placed into a new 2 ml collection tube, 100 μl of Solution C6 was added to the center of the filter membrane, and the filter was centrifuged at 10,000×*g* for 30 s at RT. The spin filter was discarded. The DNA in the tube was used for subsequent experiments. The bacterial 16S rRNA gene was amplified by qPCR using universal primers; the amplified 16S rRNA, which was normalized to glyceraldehyde 3‐phosphate dehydrogenase (GAPDH) expression level, was proportional to the number of intestinal bacteria present.

### 4.4 RNA extraction and quantitative real‐time PCR

Two‐millimeter‐cubed portions of frozen liver or 1–2 mm portions of the colon were used to obtain total RNA using TRIzol Reagent (Invitrogen) according to the manufacturer's instructions. The individual portions were ground at a low temperature in 1 ml TRIzol Reagent and were placed at RT for 5 min. Then, 200 μl of trichloromethane was added, and the tubes were vigorously vortexed, placed at RT for 3 min, and centrifuged at 12,000×*g* for 15 min at 4°C. The supernatant was carefully transferred to new tubes (140 μl × 2 = 280 μl), mixed with an equal volume of isopropanol, blended by gently inverting and centrifuged at 12,000×*g* for 15 min at 4°C. The supernatant was discarded, and 1 ml of 75% alcohol‐diethyl pyrocarbonate (DEPC) solution was added to rinse the sediment, followed by centrifugation at 8,000×*g* for 5 min at 4°C. The supernatant was discarded, and the pellet was air‐dried for 5–10 min followed by the addition of 30 μl DEPC to dissolve the sediment‐acquired RNA solution. Then, reverse transcription was performed using a cDNA Synthesis Kit (Roche) according to the manufacturer's instructions. Relative mRNA expression was measured by qPCR using SYBR Green PCR Master Mix (Roche). GAPDH mRNA was amplified in parallel as a reference gene. The primer sequences are shown in Table 1.

**Table 1:** Real‐time PCR primers

	*F* (5′ to 3′)	R (5′ to 3′)
Sox9	TGAAGAACGGACAAGCGGAG	CAGCTTGCACGTCGGTTTTG
Tgfb1	AGGAGACGGAATACAGGGCT	ATGTCATGGATGGTGCCCAG
ALR	AGGAACCAGCCAGACACAAG	CGCCAACGCTCATCTACTCT
IL‐6	AGGAGACTTCACAGAGGATACC	GAATTGCCATTGCACAACTCTT
TNF‐α	AGGGTCTGGGCCATAGAACT	CCACCACGCTCTTCTGTCTAC
IL‐1β	GTGTCTTTCCCGTGGACCTT	CGTCACACACCAGCAGGTTA
TWEAK	CCAGACAGAGGAAAGCCAGG	CTCACTGTCCCATCCACACC
ZO‐1	GAGCTACGCTTGCCACACTGT	TCGGATCTCCAGGAAGACACTT

### 4.5 Immunohistochemistry

Liver paraffin sections (5 μm thick) were successively dewaxed in xylene and were rehydrated in gradient alcohol solutions. A rabbit polyclonal antibody against SOX9 (ab26414, Abcam), an anti‐pan‐CK polyclonal rabbit antibody (Z0622, Dako), a rabbit polyclonal antibody against CD68 (ab125212) and a rabbit polyclonal antibody against ZO‐1 (40‐2200, Invitrogen) were applied (1:200), and the slides were incubated overnight at 4°C followed by rinsing with PBS. The liver sections were then incubated with a secondary antibody (PV‐6001, ZSbio, Beijing, China) for 1 hr, and the antibody was detected using DAB. The sections were counterstained in hematoxylin for 15 s, washed with tap water, dehydrated and mounted using neutral balsam. The results were analyzed by an independent, trained observer who was blinded to the experimental treatment conditions, and both the number of cells and the total density of the immunoreactive material were determined using ImageJ software.

### 4.6 Statistical analysis

Each result is presented as the mean ± SEM of at least three independent experiments. The differences between the two groups were analyzed using Student's *t* test. Statistical significance was calculated using SPSS package version 17.0, with *p* < 0.05 considered to represent statistical significance.

## CONFLICT OF INTERESTS

The authors declare no competing financial interests.

## AUTHOR CONTRIBUTIONS

Jianbo Xiu and Qi Xu conceived and designed the study. Fei Wang performed the main experiments and analyzed the data. Nan‐nan Sun, Lan‐lan Li, and Wan‐wan Zhu participated in the animal treatment and sample collection. Fei Wang wrote the manuscript. Jianbo Xiu and Yan Shen revised the manuscript.

## ETHICS STATEMENT

All animal procedures were conducted in accordance with the institutional guidelines of the Beijing Administration Office of Laboratory Animals and with the approval of the Experimental Animal Center of the Chinese Academy of Medical Sciences and Peking Union Medical College.

## Data Availability

All data are provided in full in the results section of this paper and available from the corresponding author on reasonable request.

## References

[mbo3873-bib-0001] Akahori, H. , Karmali, V. , Polavarapu, R. , Lyle, A. N. , Weiss, D. , Shin, E. , … Finn, A. V. (2015). CD163 interacts with TWEAK to regulate tissue regeneration after ischaemic injury. Nature Communications, 6, 7792 10.1038/ncomms8792 PMC4918310 26242746

[mbo3873-bib-0002] Akhurst, B. , Croager, E. J. , Farley‐Roche, C. A. , Ong, J. K. , Dumble, M. L. , Knight, B. , & Yeoh, G. C. (2001). A modified choline‐deficient, ethionine‐supplemented diet protocol effectively induces oval cells in mouse liver. Hepatology, 34, 519–522. 10.1053/jhep.2001.26751 11526537

[mbo3873-bib-0003] Bajaj, J. S. , Hylemon, P. B. , Ridlon, J. M. , Heuman, D. M. , Daita, K. , White, M. B. , … Gillevet, P. M. (2012). Colonic mucosal microbiome differs from stool microbiome in cirrhosis and hepatic encephalopathy and is linked to cognition and inflammation. American Journal of Physiology‐Gastrointestinal and Liver Physiology, 303, G675–G685. 10.1152/ajpgi.00152.2012 22821944 PMC3468538

[mbo3873-bib-0004] Barak, V. , Goike, H. , Panaretakis, K. W. , & Einarsson, R. (2004). Clinical utility of cytokeratins as tumor markers. Clinical Biochemistry, 37, 529–540. 10.1016/j.clinbiochem.2004.05.009 15234234

[mbo3873-bib-0005] Bawa, M. , & Saraswat, V. A. (2013). Gut‐liver axis: Role of inflammasomes. Journal of Clinical and Experimental Hepatology, 3, 141–149. 10.1016/j.jceh.2013.03.225 25755488 PMC4216435

[mbo3873-bib-0006] Brockmoller, J. , & Roots, I. (1994). Assessment of liver metabolic function. Clinical implications. Clinical Pharmacokinetics, 27, 216–248. 10.2165/00003088-199427030-00005 7988103

[mbo3873-bib-0007] Carotti, S. , Guarino, M. P. , Vespasiani‐Gentilucci, U. , & Morini, S. (2015). Starring role of toll‐like receptor‐4 activation in the gut‐liver axis. World Journal of Gastrointestinal Pathophysiology, 6, 99–109. 10.4291/wjgp.v6.i4.99 26600967 PMC4644892

[mbo3873-bib-0008] Chen, F. , Fang, J. , Wang, H. , Song, T. , Zhu, W. , Wu, M. , & Wu, Y. (2018). Effects of nutritional support on short‐term clinical outcomes and immune response in unresectable locally advanced oesophageal squamous cell carcinoma. European Journal of Cancer Care, 27, e12818 10.1111/ecc.12818 29345017

[mbo3873-bib-0009] Chen, Y. , Yang, F. , Lu, H. , Wang, B. , Chen, Y. , Lei, D. , … Li, L. (2011). Characterization of fecal microbial communities in patients with liver cirrhosis. Hepatology, 54, 562–572. 10.1002/hep.24423 21574172

[mbo3873-bib-0010] Chien, C.‐S. , Chen, Y.‐H. , Chen, H.‐L. , Wang, C.‐P. , Wu, S.‐H. , Ho, S.‐L. , … Chang, M.‐H. (2018). Cells responsible for liver mass regeneration in rats with 2‐acetylaminofluorene/partial hepatectomy injury. Journal of Biomedical Science, 25, 39 10.1186/s12929-018-0441-5 29695258 PMC5937839

[mbo3873-bib-0011] Chung, K. W. , Lee, E. K. , Kim, D. H. , An, H. J. , Kim, N. D. , Im, D. S. , … Chung, H. Y. (2015). Age‐related sensitivity to endotoxin‐induced liver inflammation: Implication of inflammasome/IL‐1beta for steatohepatitis. Aging Cell, 14, 524–533. 10.1111/acel.12305 25847140 PMC4531067

[mbo3873-bib-0012] Cressman, D. E. , Greenbaum, L. E. , DeAngelis, R. A. , Ciliberto, G. , Furth, E. E. , Poli, V. , & Taub, R. (1996). Liver failure and defective hepatocyte regeneration in interleukin‐6‐deficient mice. Science, 274, 1379–1383. 10.1126/science.274.5291.1379 8910279

[mbo3873-bib-0013] Feng, S.‐L. , Guo, Y. , Factor, V. M. , Thorgeirsson, S. S. , Bell, D. W. , Testa, J. R. , … Winkles, J. A. (2000). The Fn14 immediate‐early response gene is induced during liver regeneration and highly expressed in both human and murine hepatocellular carcinomas. The American Journal of Pathology, 156, 1253–1261. 10.1016/S0002-9440(10)64996-6 10751351 PMC1876890

[mbo3873-bib-0014] Fröhlich, E. E. , Farzi, A. , Mayerhofer, R. , Reichmann, F. , Jačan, A. , Wagner, B. , … Holzer, P. (2016). Cognitive impairment by antibiotic‐induced gut dysbiosis: Analysis of gut microbiota‐brain communication. Brain, Behavior, and Immunity, 56, 140–155. 10.1016/j.bbi.2016.02.020 PMC5014122 26923630

[mbo3873-bib-0015] Fukui, H. , Brauner, B. , Bode, J. C. , & Bode, C. (1991). Plasma endotoxin concentrations in patients with alcoholic and non‐alcoholic liver disease: Reevaluation with an improved chromogenic assay. Journal of Hepatology, 12, 162–169. 10.1016/0168-8278(91)90933-3 2050995

[mbo3873-bib-0016] Furuyama, K. , Kawaguchi, Y. , Akiyama, H. , Horiguchi, M. , Kodama, S. , Kuhara, T. , … Uemoto, S. (2011). Continuous cell supply from a Sox9‐expressing progenitor zone in adult liver, exocrine pancreas and intestine. Nature Genetics, 43, 34–41. 10.1038/ng.722 21113154

[mbo3873-bib-0017] Gandhi, C. R. (2012). Augmenter of liver regeneration. Fibrogenesis & Tissue Repair, 5, 10 10.1186/1755-1536-5-10 22776437 PMC3519801

[mbo3873-bib-0018] Ghosh, S. S. , Bie, J. , Wang, J. , & Ghosh, S. (2014). Oral supplementation with non‐absorbable antibiotics or curcumin attenuates western diet‐induced atherosclerosis and glucose intolerance in LDLR‐/‐ mice–role of intestinal permeability and macrophage activation. PLoS ONE, 9, e108577 10.1371/journal.pone.0108577 25251395 PMC4177397

[mbo3873-bib-0019] Giardino, A. , Innamorati, G. , Ugel, S. , Perbellini, O. , Girelli, R. , Frigerio, I. , … Bassi, C. (2017). Immunomodulation after radiofrequency ablation of locally advanced pancreatic cancer by monitoring the immune response in 10 patients. Pancreatology, 17, 962–966. 10.1016/j.pan.2017.09.008 29037917

[mbo3873-bib-0020] González, F. B. , Villar, S. R. , Toneatto, J. , Pacini, M. F. , Márquez, J. , D’Attilio, L. , … Pérez, A. R. (2018). Immune response triggered by Trypanosoma cruzi infection strikes adipose tissue homeostasis altering lipid storage, enzyme profile and adipokine expression. Medical Microbiology and Immunology. 10.1007/s00430-018-0572-z [Epub ahead of print] 30413884

[mbo3873-bib-0021] Grzelak, C. A. , Martelotto, L. G. , Sigglekow, N. D. , Patkunanathan, B. , Ajami, K. , Calabro, S. R. , … McCaughan, G. W. (2014). The intrahepatic signalling niche of hedgehog is defined by primary cilia positive cells during chronic liver injury. Journal of Hepatology, 60, 143–151. 10.1016/j.jhep.2013.08.012 23978713

[mbo3873-bib-0022] Gu, L. , Dai, L. , Cao, C. , Zhu, J. , Ding, C. , Xu, H. B. , … Di, W. (2013). Functional expression of TWEAK and the receptor Fn14 in human malignant ovarian tumors: Possible implication for ovarian tumor intervention. PLoS ONE, 8, e57436 10.1371/journal.pone.0057436 23469193 PMC3587594

[mbo3873-bib-0023] Guida, F. , Turco, F. , Iannotta, M. , De Gregorio, D. , Palumbo, I. , Sarnelli, G. , … Maione, S. (2018). Antibiotic‐induced microbiota perturbation causes gut endocannabinoidome changes, hippocampal neuroglial reorganization and depression in mice. Brain, Behavior, and Immunity, 67, 230–245. 10.1016/j.bbi.2017.09.001 28890155

[mbo3873-bib-0024] Hamill, C. A. , Michaelson, J. S. , Hahm, K. , Burkly, L. C. , & Kessler, J. A. (2007). Age‐dependent effects of TWEAK/Fn14 receptor activation on neural progenitor cells. Journal of Neuroscience Research, 85, 3535–3544. 10.1002/jnr.21443 17803219

[mbo3873-bib-0025] Hao, P. P. , Lee, M. J. , Yu, G. R. , Kim, I. H. , Cho, Y. G. , & Kim, D. G. (2013). Isolation of EpCAM(+)/CD133(‐) hepatic progenitor cells. Molecules and Cells, 36, 424–431. 10.1007/s10059-013-0190-y 24293012 PMC3887933

[mbo3873-bib-0026] Heimesaat, M. M. , Bereswill, S. , Fischer, A. , Fuchs, D. , Struck, D. , Niebergall, J. , … Liesenfeld, O. (2006). Gram‐negative bacteria aggravate murine small intestinal Th1‐type immunopathology following oral infection with Toxoplasma gondii. The Journal of Immunology, 177, 8785–8795. 10.4049/jimmunol.177.12.8785 17142781

[mbo3873-bib-0027] Heldin, C. H. , Miyazono, K. , & Ten, D. P. (1997). TGF‐beta signalling from cell membrane to nucleus through SMAD proteins. Nature, 390, 465–471. 9393997 10.1038/37284

[mbo3873-bib-0028] Hongbo, S. , Yu, C. , Ming, K. , Honglin, S. , Ping, H. Y. , & Ping, D. Z. (2012). Augmenter of liver regeneration may be a candidate for prognosis of HBV related acute‐on‐chronic liver failure as a regenerative marker. Hepato‐Gastroenterology, 59, 1933–1938. 10.5754/hge11679 22246190

[mbo3873-bib-0029] Huch, M. , Dorrell, C. , Boj, S. F. , van Es, J. H. , Li, V. S. W. , van de Wetering, M. , … Clevers, H. (2013). In vitro expansion of single Lgr5+ liver stem cells induced by Wnt‐driven regeneration. Nature, 494, 247–250. 10.1038/nature11826 23354049 PMC3634804

[mbo3873-bib-0030] Imajo, K. , Fujita, K. , Yoneda, M. , Nozaki, Y. , Ogawa, Y. , Shinohara, Y. , … Nakajima, A. (2012). Hyperresponsivity to low‐dose endotoxin during progression to nonalcoholic steatohepatitis is regulated by leptin‐mediated signaling. Cell Metabolism, 16, 44–54. 10.1016/j.cmet.2012.05.012 22768838

[mbo3873-bib-0031] Irie, T. , Asahina, K. , Shimizu‐Saito, K. , Teramoto, K. , Arii, S. , & Teraoka, H. (2007). Hepatic progenitor cells in the mouse extrahepatic bile duct after a bile duct ligation. Stem Cells and Development, 16, 979–987. 10.1089/scd.2007.0037 18004941

[mbo3873-bib-0032] Ivanov, I. I. , & Honda, K. (2012). Intestinal commensal microbes as immune modulators. Cell Host & Microbe, 12, 496–508. 10.1016/j.chom.2012.09.009 23084918 PMC3516493

[mbo3873-bib-0033] Jakubowski, A. , Ambrose, C. , Parr, M. , Lincecum, J. M. , Wang, M. Z. , Zheng, T. S. , … Burkly, L. C. (2005). TWEAK induces liver progenitor cell proliferation. Journal of Clinical Investigation, 115, 2330–2340. 10.1172/JCI23486 16110324 PMC1187931

[mbo3873-bib-0034] Jang, J. W. , Oh, B. S. , Kwon, J. H. , You, C. R. , Chung, K. W. , Kay, C. S. , & Jung, H. S. (2012). Serum interleukin‐6 and C‐reactive protein as a prognostic indicator in hepatocellular carcinoma. Cytokine, 60, 686–693. 10.1016/j.cyto.2012.07.017 22906998

[mbo3873-bib-0035] Ji, T. , Li, G. , Chen, J. , Zhao, J. , Li, X. I. , Lin, H. , … Cang, Y. (2016). Distinct role of interleukin‐6 and tumor necrosis factor receptor‐1 in oval cell‐mediated liver regeneration and inflammation‐associated hepatocarcinogenesis. Oncotarget, 7, 66635–66646. 10.18632/oncotarget.11365 27556180 PMC5341826

[mbo3873-bib-0036] Jinkawa, A. , Shimizu, M. , Nishida, K. , Kaneko, S. , Usami, M. , Sakumura, N. , … Yachie, A. (2019). Cytokine profile of macrophage activation syndrome associated with Kawasaki disease. Cytokine, 119, 52–56. 10.1016/j.cyto.2019.03.001 30877950

[mbo3873-bib-0037] Jones, S. A. , Horiuchi, S. , Topley, N. , Yamamoto, N. , & Fuller, G. M. (2001). The soluble interleukin 6 receptor: Mechanisms of production and implications in disease. The FASEB Journal, 15, 43–58. 10.1096/fj.99-1003rev 11149892

[mbo3873-bib-0038] Kiraly, D. D. , Walker, D. M. , Calipari, E. S. , Labonte, B. , Issler, O. , Pena, C. J. , … Nestler, E. J. (2016). Alterations of the host microbiome affect behavioral responses to cocaine. Scientific Reports, 6, 35455 10.1038/srep35455 27752130 PMC5067576

[mbo3873-bib-0039] Knell, A. J. (1980). Liver function and failure: The evolution of liver physiology. Journal of the Royal College of Physicians of London, 14, 205–208. 7009850 PMC5373221

[mbo3873-bib-0040] Köhn‐Gaone, J. , Dwyer, B. J. , Grzelak, C. A. , Miller, G. , Shackel, N. A. , Ramm, G. A. , … Tirnitz‐Parker, J. E. E. (2016). Divergent inflammatory, fibrogenic, and liver progenitor cell dynamics in two common mouse models of chronic liver injury. The American Journal of Pathology, 186, 1762–1774. 10.1016/j.ajpath.2016.03.005 27181403

[mbo3873-bib-0041] Krarup, N. , Larsen, J. A. , & Madsen, B. (1975). Liver function, liver hemodynamics and intrahepatic distribution of portal blood in cast during slight hypothermia. Acta Physiologica Scandinavica, 93, 566–568. 1155153 10.1111/j.1748-1716.1975.tb05851.x

[mbo3873-bib-0042] Kubo, M. , Hanada, T. , & Yoshimura, A. (2003). Suppressors of cytokine signaling and immunity. Nature Immunology, 4, 1169–1176. 10.1038/ni1012 14639467

[mbo3873-bib-0043] Levy, D. E. , & Lee, C. K. (2002). What does Stat3 do? Journal of Clinical Investigation, 109, 1143–1148. 10.1172/JCI0215650 11994402 PMC150972

[mbo3873-bib-0044] Li, J. , Wu, W. , Xin, J. , Guo, J. , Jiang, L. , Tao, R. , … Li, L. (2011). Acute hepatic failure‐derived bone marrow mesenchymal stem cells express hepatic progenitor cell genes. Cells Tissues Organs, 194, 371–381. 10.1159/000322604 21293100

[mbo3873-bib-0045] Li, W.‐L. , Su, J. , Yao, Y.‐C. , Tao, X.‐R. , Yan, Y.‐B. , Yu, H.‐Y. , … Hu, Y.‐P. (2006). Isolation and characterization of bipotent liver progenitor cells from adult mouse. Stem Cells, 24, 322–332. 10.1634/stemcells.2005-0108 16109753

[mbo3873-bib-0046] Liu, D. , Yovchev, M. I. , Zhang, J. , Alfieri, A. A. , Tchaikovskaya, T. , Laconi, E. , & Dabeva, M. D. (2015). Identification and characterization of mesenchymal‐epithelial progenitor‐like cells in normal and injured rat liver. The American Journal of Pathology, 185, 110–128. 10.1016/j.ajpath.2014.08.029 25447047 PMC4278240

[mbo3873-bib-0047] Liu, Q. , Xiao, S. , & Xia, Y. (2017). TWEAK/Fn14 activation participates in skin inflammation. Mediators of Inflammation, 2017, 6746870 10.1155/2017/6746870 29038621 PMC5606047

[mbo3873-bib-0048] Lo, R. C. , Chan, K. K. , Leung, C. O. , & Ng, I. O. (2018). Expression of hepatic progenitor cell markers in acute cellular rejection of liver allografts‐An immunohistochemical study. Clinical Transplantation, 32, e13203 10.1111/ctr.13203 29345755

[mbo3873-bib-0049] Lu, H. , Wu, Z. , Xu, W. , Yang, J. , Chen, Y. , & Li, L. (2011). Intestinal microbiota was assessed in cirrhotic patients with hepatitis B virus infection. Intestinal microbiota of HBV cirrhotic patients. Microbial Ecology, 61, 693–703. 10.1007/s00248-010-9801-8 21286703

[mbo3873-bib-0050] Lu, J. , Zhou, Y. , Hu, T. , Zhang, H. , Shen, M. , Cheng, P. , … Guo, C. (2016). Notch signaling coordinates progenitor cell‐mediated biliary regeneration following partial hepatectomy. Scientific Reports, 6, 22754 10.1038/srep22754 26951801 PMC4782135

[mbo3873-bib-0051] Lu, W.‐Y. , Bird, T. G. , Boulter, L. , Tsuchiya, A. , Cole, A. M. , Hay, T. , … Forbes, S. J. (2015). Hepatic progenitor cells of biliary origin with liver repopulation capacity. Nature Cell Biology, 17, 971–983. 10.1038/ncb3203 26192438 PMC4612439

[mbo3873-bib-0052] Lundberg, R. , Toft, M. F. , August, B. , Hansen, A. K. , & Hansen, C. H. (2016). Antibiotic‐treated versus germ‐free rodents for microbiota transplantation studies. Gut Microbes, 7, 68–74. 10.1080/19490976.2015.1127463 26744774 PMC4856451

[mbo3873-bib-0053] Malaguarnera, G. , Giordano, M. , Nunnari, G. , Bertino, G. , & Malaguarnera, M. (2014). Gut microbiota in alcoholic liver disease: Pathogenetic role and therapeutic perspectives. World Journal of Gastroenterology, 20, 16639–16648. 10.3748/wjg.v20.i44.16639 25469033 PMC4248208

[mbo3873-bib-0054] Mendlovic, F. , Cruz‐Rivera, M. , Diaz‐Gandarilla, J. A. , Flores‐Torres, M. A. , Avila, G. , Perfiliev, M. , … Flisser, A. (2017). Orally administered Taenia solium Calreticulin prevents experimental intestinal inflammation and is associated with a type 2 immune response. PLoS ONE, 12, e186510 10.1371/journal.pone.0186510 PMC5643116 29036211

[mbo3873-bib-0055] Milani, C. , Duranti, S. , Bottacini, F. , Casey, E. , Turroni, F. , Mahony, J. , … Ventura, M. (2017). The first microbial colonizers of the human gut: composition, activities, and health implications of the infant gut microbiota. Microbiology and Molecular Biology Reviews, 81 10.1128/MMBR.00036-17 PMC5706746 29118049

[mbo3873-bib-0056] Miura, K. , & Ohnishi, H. (2014). Role of gut microbiota and Toll‐like receptors in nonalcoholic fatty liver disease. World Journal of Gastroenterology, 20, 7381–7391. 10.3748/wjg.v20.i23.7381 24966608 PMC4064083

[mbo3873-bib-0057] Miyake, Y. , & Yamamoto, K. (2013). Role of gut microbiota in liver diseases. Hepatology Research, 43, 139–146. 10.1111/j.1872-034X.2012.01088.x 22970713 PMC3894231

[mbo3873-bib-0058] Möhle, L. , Mattei, D. , Heimesaat, M. M. , Bereswill, S. , Fischer, A. , Alutis, M. , … Wolf, S. A. (2016). Ly6C(hi) monocytes provide a link between antibiotic‐induced changes in gut microbiota and adult hippocampal neurogenesis. Cell Reports, 15, 1945–1956. 10.1016/j.celrep.2016.04.074 27210745

[mbo3873-bib-0059] Moll, R. (1998). Cytokeratins as markers of differentiation in the diagnosis of epithelial tumors. SubCellular Biochemistry, 31, 205–262. 9932494

[mbo3873-bib-0060] Morell, C. M. , Fiorotto, R. , Meroni, M. , Raizner, A. , Torsello, B. , Cadamuro, M. , … Strazzabosco, M. (2017). Notch signaling and progenitor/ductular reaction in steatohepatitis. PLoS ONE, 12, e187384 10.1371/journal.pone.0187384 PMC5687773 29140985

[mbo3873-bib-0061] Mu, M. , Zhang, Z. , Cheng, Y. I. , Liu, G. , Chen, X. , Wu, X. , … You, S. (2016). Augmenter of liver regeneration (ALR) restrains concanavalin A‐induced hepatitis in mice. International Immunopharmacology, 35, 280–286. 10.1016/j.intimp.2016.03.040 27085679

[mbo3873-bib-0062] Mu, Y. P. , Zhang, X. , Xu, Y. , Fan, W. W. , Li, X. W. , Chen, J. M. , … Liu, P. (2017). Notch signaling pathway participates in the differentiation of hepatic progenitor cells into bile duct epithelial cells and progression of hepatic fibrosis in cholestatic liver fibrosis rat. Zhonghua Bing Li Xue Za Zhi, 46, 400–405. 10.3760/cma.j.issn.0529-5807.2017.06.007 28591987

[mbo3873-bib-0063] Naseem, S. , Hussain, T. , & Manzoor, S. (2018). Interleukin‐6: A promising cytokine to support liver regeneration and adaptive immunity in liver pathologies. Cytokine & Growth Factor Reviews, 39, 36–45. 10.1016/j.cytogfr.2018.01.002 29361380

[mbo3873-bib-0064] Nicolini, A. , Ferrari, P. , & Rossi, G. (2015). Mucins and cytokeratins as serum tumor markers in breast cancer. Advances in Experimental Medicine and Biology, 867, 197–225. 10.1007/978-94-017-7215-0_13 26530368

[mbo3873-bib-0065] Orrhage, K. , & Nord, C. E. (2000). Bifidobacteria and lactobacilli in human health. Drugs under Experimental and Clinical Research, 26, 95–111. 10941602

[mbo3873-bib-0066] Passman, A. M. , Strauss, R. P. , McSpadden, S. B. , Finch‐Edmondson, M. L. , Woo, K. H. , Diepeveen, L. A. , … Yeoh, G. C. (2015). A modified choline‐deficient, ethionine‐supplemented diet reduces morbidity and retains a liver progenitor cell response in mice. Disease Models & Mechanisms, 8, 1635–1641. 10.1242/dmm.022020 26496771 PMC4728320

[mbo3873-bib-0067] Petersen, B. E. , Zajac, V. F. , & Michalopoulos, G. K. (1998). Hepatic oval cell activation in response to injury following chemically induced periportal or pericentral damage in rats. Hepatology, 27, 1030–1038. 10.1002/hep.510270419 9537443

[mbo3873-bib-0068] Pozniak, K. N. , Pearen, M. A. , Pereira, T. N. , Kramer, C. S. M. , Kalita‐De Croft, P. , Nawaratna, S. K. , … Ramm, G. A. (2017). Taurocholate induces biliary differentiation of liver progenitor cells causing hepatic stellate cell chemotaxis in the ductular reaction: Role in pediatric cystic fibrosis liver disease. The American Journal of Pathology, 187, 2744–2757. 10.1016/j.ajpath.2017.08.024 28935574

[mbo3873-bib-0069] Qin, N. , Yang, F. , Li, A. , Prifti, E. , Chen, Y. , Shao, L. I. , … Li, L. (2014). Alterations of the human gut microbiome in liver cirrhosis. Nature, 513, 59–64. 10.1038/nature13568 25079328

[mbo3873-bib-0070] Raven, A. , Lu, W. Y. , Man, T. Y. , Ferreira‐Gonzalez, S. , O'Duibhir, E. , Dwyer, B. J. , … Forbes, S. J. (2018). Corrigendum: Cholangiocytes act as facultative liver stem cells during impaired hepatocyte regeneration. Nature, 555, 402. 10.1038/nature25996 29542689

[mbo3873-bib-0071] Rincon, M. (2012). Interleukin‐6: From an inflammatory marker to a target for inflammatory diseases. Trends in Immunology, 33, 571–577. 10.1016/j.it.2012.07.003 22883707

[mbo3873-bib-0072] Rivera, C. A. , Bradford, B. U. , Seabra, V. , & Thurman, R. G. (1998). Role of endotoxin in the hypermetabolic state after acute ethanol exposure. American Journal of Physiology‐Gastrointestinal and Liver Physiology, 275, G1252–G1258. 10.1152/ajpgi.1998.275.6.G1252 9843760

[mbo3873-bib-0073] Russell, J. O. , Lu, W. Y. , Okabe, H. , Abrams, M. , Oertel, M. , Poddar, M. , … Monga, S. P. (2019). Hepatocyte‐specific β‐catenin deletion during severe liver injury provokes cholangiocytes to differentiate into hepatocytes. Hepatology, 69, 742–759. 30215850 10.1002/hep.30270 PMC6351199

[mbo3873-bib-0074] Sato, A. , Nakashima, H. , Nakashima, M. , Ikarashi, M. , Nishiyama, K. , Kinoshita, M. , & Seki, S. (2014). Involvement of the TNF and FasL produced by CD11b Kupffer cells/macrophages in CCl4‐induced acute hepatic injury. PLoS ONE, 9, e92515 10.1371/journal.pone.0092515 24667392 PMC3965417

[mbo3873-bib-0075] Seabra, V. , Stachlewitz, R. F. , & Thurman, R. G. (1998). Taurine Blunts LPS-induced Increases in Intracellular Calcium and TNF-alpha Production by Kupffer Cells. Journal of Leukocyte Biology, 64, 615–621. 9823766 10.1002/jlb.64.5.615

[mbo3873-bib-0076] Seoane, J. , & Gomis, R. R. (2017). TGF‐β family signaling in tumor suppression and cancer progression. Cold Spring Harbor Perspectives in Biology, 9 :a022277 10.1101/cshperspect.a022277 28246180 PMC5710110

[mbo3873-bib-0077] Sheng, Z. , Ju, C. , Li, B. , Chen, Z. , Pan, X. , Yan, G. , … Ma, G. (2018). TWEAK promotes endothelial progenitor cell vasculogenesis to alleviate acute myocardial infarction via the Fn14‐NF‐kappaB signaling pathway. Experimental and Therapeutic Medicine, 16, 4019–4029. 30344680 10.3892/etm.2018.6703 PMC6176210

[mbo3873-bib-0078] Shi, Y. , & Massague, J. (2003). Mechanisms of TGF‐beta signaling from cell membrane to the nucleus. Cell, 113, 685–700. 10.1016/s0092-8674(03)00432-x 12809600

[mbo3873-bib-0079] Shrivastava, S. , Mukherjee, A. , Ray, R. , & Ray, R. B. (2013). Hepatitis C virus induces interleukin‐1beta (IL‐1beta)/IL‐18 in circulatory and resident liver macrophages. Journal of Virology, 87, 12284–12290. 10.1128/jvi.01962-13 24006444 PMC3807883

[mbo3873-bib-0080] Suzuki, A. , Sekiya, S. , Onishi, M. , Oshima, N. , Kiyonari, H. , Nakauchi, H. , & Taniguchi, H. (2008). Flow cytometric isolation and clonal identification of self‐renewing bipotent hepatic progenitor cells in adult mouse liver. Hepatology, 48, 1964–1978. 10.1002/hep.22558 18837044

[mbo3873-bib-0081] Szabo, G. (2015). Gut‐liver axis in alcoholic liver disease. Gastroenterology, 148, 30–36. 10.1053/j.gastro.2014.10.042 25447847 PMC4274189

[mbo3873-bib-0082] Tanaka, M. , & Nakayama, J. (2017). Development of the gut microbiota in infancy and its impact on health in later life. Allergology International, 66, 515–522. 10.1016/j.alit.2017.07.010 28826938

[mbo3873-bib-0083] Tanaka, T. , & Kishimoto, T. (2014). The biology and medical implications of interleukin‐6. Cancer Immunology Research, 2, 288–294. 10.1158/2326-6066.CIR-14-0022 24764575

[mbo3873-bib-0084] Tanimizu, N. , Nishikawa, Y. , Ichinohe, N. , Akiyama, H. , & Mitaka, T. (2014). Sry HMG box protein 9‐positive (Sox9+) epithelial cell adhesion molecule‐negative (EpCAM‐) biphenotypic cells derived from hepatocytes are involved in mouse liver regeneration. Journal of Biological Chemistry, 289, 7589–7598. 10.1074/jbc.m113.517243 24482234 PMC3953272

[mbo3873-bib-0085] Tarlow, B. D. , Finegold, M. J. , & Grompe, M. (2014). Clonal Tracing of Sox9+ Liver Progenitors in Mouse Oval Cell Injury. Hepatology, 60, 278–289. 24700457 10.1002/hep.27084 PMC4077948

[mbo3873-bib-0086] Tee, L. B. , Kirilak, Y. , Huang, W. H. , Morgan, R. H. , & Yeoh, G. C. (1994). Differentiation of oval cells into duct‐like cells in preneoplastic liver of rats placed on a choline‐deficient diet supplemented with ethionine. Carcinogenesis, 15, 2747–2756. 10.1093/carcin/15.12.2747 8001230

[mbo3873-bib-0087] Tee, L. B. , Smith, P. G. , & Yeoh, G. C. (1992). Expression of alpha, mu and pi class glutathione S‐transferases in oval and ductal cells in liver of rats placed on a choline‐deficient, ethionine‐supplemented diet. Carcinogenesis, 13, 1879–1885. 10.1093/carcin/13.10.1879 1423848

[mbo3873-bib-0088] Tirnitz‐Parker, J. E. , Viebahn, C. S. , Jakubowski, A. , Klopcic, B. R. , Olynyk, J. K. , Yeoh, G. C. , & Knight, B. (2010). Tumor necrosis factor‐like weak inducer of apoptosis is a mitogen for liver progenitor cells. Hepatology, 52, 291–302. 10.1002/hep.23663 20578156

[mbo3873-bib-0089] Tuomisto, S. , Pessi, T. , Collin, P. , Vuento, R. , Aittoniemi, J. , & Karhunen, P. J. (2014). Changes in gut bacterial populations and their translocation into liver and ascites in alcoholic liver cirrhotics. BMC Gastroenterology, 14, 40 10.1186/1471-230X-14-40 24564202 PMC3996058

[mbo3873-bib-0090] Vodovotz, Y. , Prelich, J. , Lagoa, C. , Barclay, D. , Zamora, R. , Murase, N. , & Gandhi, C. R. (2013). Augmenter of liver regeneration (ALR) is a novel biomarker of hepatocellular stress/inflammation: In vitro, in vivo and in silico studies. Molecular Medicine, 18, 1421–1429. 10.2119/molmed.2012.00183 23073658 PMC3563711

[mbo3873-bib-0091] Wang, A. , Zhang, F. , Xu, H. , Xu, M. , Cao, Y. , Wang, C. , … Zhuge, Y. (2017). TWEAK/Fn14 promotes pro‐inflammatory cytokine secretion in hepatic stellate cells via NF‐kappaB/STAT3 pathways. Molecular Immunology, 87, 67–75. 10.1016/j.molimm.2017.04.003 28411440

[mbo3873-bib-0092] Wilson, J. W. , & Leduc, E. H. (1958). Role of cholangioles in restoration of the liver of the mouse after dietary injury. The Journal of Pathology and Bacteriology, 76, 441–449. 10.1002/path.1700760213 13588479

[mbo3873-bib-0093] Wu, H. H. , & Lee, O. K. (2017). Exosomes from mesenchymal stem cells induce the conversion of hepatocytes into progenitor oval cells. Stem Cell Research & Therapy, 8, 117 10.1186/s13287-017-0560-z 28535778 PMC5442870

[mbo3873-bib-0094] Wu, M. Y. , & Hill, C. S. (2009). TGF‐β superfamily signaling in embryonic development and homeostasis. Developmental Cell, 16, 329–343. 10.1016/j.devcel.2009.02.012 19289080

[mbo3873-bib-0095] Wu, P. C. , Ma, L. , Gibson, J. B. , Hirai, H. , & Tsukada, Y. (1981). Serum alpha‐fetoprotein in rats after ligation of the common bile duct: Relation to ductular cell (oval cell) proliferation. The Journal of Pathology, 133, 61–74. 10.1002/path.1711330107 6162935

[mbo3873-bib-0096] Xu, W. D. , Zhao, Y. , & Liu, Y. (2016). Role of the TWEAK/Fn14 pathway in autoimmune diseases. Immunologic Research, 64, 44–50. 10.1007/s12026-015-8761-y 26659091

[mbo3873-bib-0097] Yamada, S. , Kamada, N. , Amiya, T. , Nakamoto, N. , Nakaoka, T. , Kimura, M. , … Saito, H. (2017). Gut microbiota‐mediated generation of saturated fatty acids elicits inflammation in the liver in murine high‐fat diet‐induced steatohepatitis. BMC Gastroenterology, 17, 136 10.1186/s12876-017-0689-3 29187142 PMC5708095

[mbo3873-bib-0098] Yang, X. , He, F. , Zhang, Y. , Xue, J. , Li, K. , Zhang, X. , … Shaoqi Y. (2019). Inulin ameliorates alcoholic liver disease via suppressing LPS‐TLR4‐Mψ axis and modulating gut microbiota in mice. Alcoholism, Clinical and Experimental Research, 43, 411–424. 10.1111/acer.13950 30589437

[mbo3873-bib-0099] Yeoh, G. C. T. , Ernst, M. , Rose‐John, S. , Akhurst, B. , Payne, C. , Long, S. , … Matthews, V. B. (2007). Opposing roles of gp130‐mediated STAT‐3 and ERK‐1/ 2 signaling in liver progenitor cell migration and proliferation. Hepatology, 45, 486–494. 10.1002/hep.21535 17256754

[mbo3873-bib-0100] Zhai, R. , Wang, Y. , Qi, L. E. , Williams, G. M. , Gao, B. , Song, G. , … Sun, Z. (2018). Pharmacological mobilization of endogenous bone marrow stem cells promotes liver regeneration after extensive liver resection in rats. Scientific Reports, 8, 3587 10.1038/s41598-018-21961-2 29483616 PMC5827664

